# Simultaneous Bilateral Cochlear Implantation in Adults

**DOI:** 10.3390/jpm13101462

**Published:** 2023-10-04

**Authors:** Nawaf Fatani, Nezar Hamed, Abdulrahman Hagr

**Affiliations:** King Abdullah Ear Specialist Center (KAESC), College of Medicine, King Saud University Medical City (KSUMC), King Saud University, Riyadh 11411, Saudi Arabia

**Keywords:** cochlear implant, cochlear implantation, bilateral, simultaneous, adults

## Abstract

The objective of this study is to review our experience with simultaneous bilateral cochlear implantation (BiCI) in adults, and assess its feasibility. This could shorten the time required to regain binaural hearing, prevent social isolation, and potentially eliminate the need for hearing aids, as seen with sequential BiCI. A retrospective study was conducted involving adult patients who received simultaneous BiCI at our center between 2010 and 2023. The feasibility of simultaneous BiCI was assessed through postoperative clinical evaluations, outpatient visits, discharge status, and the acceptance of device fitting. Twenty-seven patients underwent simultaneous BiCIs. Their mean age was 37 years, comprising 59.3% males and 40.7% females. Out of the included patients, 51.9% had childhood-onset hearing loss, while 29.6% developed hearing loss later in life. Causes of hearing loss included meningitis 7.4%, trauma 11.1%, non-specific high-grade fever 11.1%, and Brucellosis infection 3.7%. Labyrinthine ossificans (LO) was present in 7.4%, and retrofenestral otospongiosis in 3.7%. The post-operative period and initial outpatient visit were uneventful for 88.8% and 81.5% of patients, respectively. Intraoperative complications were absent in 96.2% of cases. Simultaneous BiCI is feasible in adults without major intraoperative complications or troublesome recovery periods, offering potential benefits by reducing the number of surgeries and hospital admissions compared to the sequential method.

## 1. Introduction

Sensorineural hearing loss (SNHL) is the third major cause of years lived with disability in adults. This type of hearing loss often worsens with age. According to the WHO, 466 million people over the age of 65 experienced hearing loss in 2018, which makes hearing loss one of the most common major reasons for disability globally [1]. Hearing impairment, apart from being a significant burden to the patient and society, is also reportedly associated with an increase in the prevalence of several other health problems (e.g., depression). Additionally, communication difficulty is one of the most significant effects of any hearing loss; lack of communication can result in feelings of isolation and frustration, which can ultimately contribute to poor mental health [2]. There is evidence showing that hearing loss is correlated with cognitive decline in elderly patients; thus, managing these patients can have a significant impact on their quality of life [3]. The first line of management is focused on prevention and rehabilitation, because there is currently no established treatment that may reverse pathological damage at the cochlear level [4]. Hearing aids are the initial management for hearing loss, but patients with severe to profound sensorineural hearing loss are unlikely to benefit from them. In these situations, cochlear implants (CIs) are the optimal option [5].

Cochlear implants (CIs) are a surgically implantable device beneath the skin and over the temporal bone with an electrode array inserted into the damaged cochlea [6]. They are considered a potential alternative to hearing aids because they replace the role of hair cells that can no longer generate electrical impulses in response to noises by transmitting these electrical impulses directly to the spiral ganglion neurons and the cochlear nerve [6,7]. Initially, only those with profound bilateral deafness were considered candidates for cochlear implants; however, as cochlear implant technology and processing strategies advanced over the years, the candidacy criteria for CI surgery were expanded for both adults and children, with a wide range of indications [8].

Unilateral cochlear implants (CI) are commonly employed as the initial treatment for severe to profound bilateral sensorineural hearing loss in adults. Although, studies have demonstrated improvements in speech perception and auditory outcomes with unilateral CIs in adults, those users frequently have more trouble understanding speech in a noisy setting, and are less able to hear sound coming from the direction of their nonimplanted ear [9]. In addition, the National Institute for Health and Clinical Excellence (NICE) advises against bilateral cochlear implantation (BiCI) in adults unless accompanied by other disabilities that make them more dependent on auditory inputs as their main sensory method for spatial awareness [10]. Furthermore, the provision of a second CI to adults is limited in some healthcare systems due to a lack of sufficient evidence regarding societal benefits [11].

Nevertheless, bilateral cochlear implantation is currently recognized as an acceptable method for hearing rehabilitation in adults [12]. There are two surgical methods for bilateral CIs; one is sequential BiCI, in which each ear has a separate operation with intervals ranging from months to years. On the contrary, in simultaneous BiCI, both implants are placed within one surgery. These types of implantations also vary in terms of surgical risks, preoperative and postoperative care, hospital stays, and financial costs [13]. It is known that binaural hearing is beneficial for normal-hearing individuals when listening to speech in noisy situations. Compared to unilateral listening, speech intelligibility in noise can improve when both ears are functioning [12]. Studies have shown that binaural hearing has several advantages in terms of sound localization, noise discrimination, speech recognition, the squelch effect, the head shadow effect, and binaural summation [9,13,14]. Among these, there are three main advantages for binaural hearing; the first is the head shadow effect due to differential sound filtering caused by the physical head. The signal-to-noise ratio (SNR) to each ear is different, so if both ears are working, the listener can focus their attention on the ear with the best SNR to improve speech recognition. Another feature is the squelch effect, in which the auditory system is able to combine the information when it receives functional input from both ears to create a more accurate representation. The use of this effect can greatly enhance speech understanding. Binaural summation is believed to occur when noise and speech come from the same place. Summation is the ability of the auditory system to aggregate and profit from multiple representations of the same information to the two ears, increasing perceived loudness as a result [9].

A recent study on adult patients found comparable objective and subjective hearing outcomes between simultaneous and sequential BiCI after one year. There was also a significant improvement in speech intelligibility in noise in the simultaneous BiCI group. Likewise, there was better performance in the sequential group with the second CI compared to the unilateral CI. However, the study did not focus on the surgical approach and clinical outcomes of simultaneous BiCI surgery [15]. Another study reported that second CI in sequential BiCI and simultaneous BiCI showed a positive significant correlation between speech discrimination score and total usage time [14]. Additionally, a survey study revealed that sequential BiCI is more commonly performed in adults, possibly due to limited counseling compared to pediatric cases. Moreover, giving bilateral simultaneous cochlear implantation to children appears to be more focused on maximizing their developmental results than on providing adults with binaural benefits; uncertainty exists whether this is related to the practice of CI centers or to healthcare system priorities [8].

Traditionally, the external sound processor is activated 4–6 weeks after cochlear implant surgery in several CI centers to allow for proper wound healing and to prevent flap infections. Moreover, there are concerns about implant migration and the instability of the electrode impedances. All these issues will delay patients in starting hearing rehabilitation, and yet there is no gold standard for the period during which to fit and activate the external device. However, advancements in surgical techniques have facilitated faster wound healing and reduced complications by utilizing smaller and different incisions, along with the continuous growth and development of these prosthesis technologies. As a result, early activation of the external sound processor a few days after CI surgery has become generally safe and feasible [16,17,18,19,20,21]. A study showed that 4 weeks of activation after CI surgery was not superior to early activation within 24 h regarding preserving residual hearing, changes in impedances, and speech perception, and no significant complication occurred in both groups [19]. Early activation offers the potential for earlier hearing experience and rehabilitation, making it an attractive option for some CI patients [18,20]. However, the benefits of simultaneous BiCI have been proven in pediatric populations in terms of safety, efficacy, and speech outcomes, and the procedure requires fewer resources for rehabilitation compared to sequential BiCI [8,22,23,24,25,26,27]. There is still a lack of evidence regarding the surgical and clinical outcomes of simultaneous BiCI in adults in achieving faster rehabilitation.

Many adult patients prefer initially to undergo CI surgery on one side and, if successful, proceed to the other side [28]. Moreover, the waiting period of up to one month without hearing after surgery makes simultaneous BiCI less appealing for patients. In our center, we have extensive experience in early activation and bilateral simultaneous CI surgery in both adults and children.

The objective of this study is to review our experience with simultaneous BiCI in adults and evaluate its feasibility in light of the aforementioned considerations. This could shorten the time required to regain binaural hearing, prevent social isolation, and potentially eliminate the need for hearing aids, as seen with sequential BiCI [19].

## 2. Materials and Methods

### 2.1. Study Design

This retrospective study encompassed all adult patients who underwent bilateral simultaneous cochlear implantation (CI) at our tertiary CI center between 2010 and 2023. Inclusion criteria comprised adults defined age as 18 and above, diagnosed with bilateral severe to profound sensorineural hearing loss, with minimal or no benefit from hearing aids. Exclusion criteria included inner ear malformations, chronic ear disease, and incomplete data. Data were collected on various characteristics, including demographic information, onset of hearing loss, risk factors, clinical evaluations, radiological images, surgical procedures and findings, surgical time (incision-suture time), postoperative recovery in terms of pain, wound status, and initial outpatient assessments. The feasibility of simultaneous bilateral cochlear implantation BiCI was assessed through postoperative clinical evaluations, outpatient visits, discharge status, and patient’s acceptance of the fitting without complaints or issues. The study protocol was reviewed and approved by the institutional review board (Reference No. 23/0133/IRB).

### 2.2. CI Surgery

All patients underwent CI surgery at our center, following a consistent surgical approach. Mastoidectomy and posterior tympanotomy were performed using the standard technique for all participants. Patients were positioned supine in the operating room. Facial nerve monitoring was conducted by stimulating both sides using two channels in the midline. No shaving was performed. Postauricular areas for both ears were sterilized using a povidone-based solution. A large drape was placed from behind the head to the mid-face anteriorly, creating a sterile field covering both ears. Local anesthesia with 1% lidocaine and 1/100,000 epinephrine was administered. A 3 cm incision, located 1 cm posterior to the auricular sulcus, was made. This limited incision technique was employed in all patients to facilitate faster wound healing. The palva flap was elevated anteriorly, followed by standard mastoidectomy and elevation of a posterosuperior flap for creating the pocket to accommodate the implant (receiver–stimulator). A posterior tympanotomy was performed with skeletonization of the facial nerve and chorda tempani. Electrode insertion through the round-window approach was performed for all patients. Closure involved the use of 3.0 vicryl sutures for the subcutaneous layer and 5.0 monocrystals for skin closure using the subcuticular technique. Electrically evoked compound action potential (ECAP) measurements were conducted bilaterally. The patient was awakened by the anesthesia team and transferred to the recovery area in a stable condition. Intraoperatively, all patients received a single dose of antibiotics (cefuroxime or cefazolin) and steroids (dexamethasone). The patient’s surgical wounds were examined on the following day to assess healing and detect any swelling, hematoma, or signs of infection.

### 2.3. Statistical Analysis

Data obtained from medical records were directly transferred to a dedicated database using Excel 2010 (Microsoft, Redmond, WA, USA). Statistical analysis was performed using SPSS v20.0 for Windows (IBM, Armonk, NY, USA). Numerical data were presented as mean ± standard deviation or as median and range, depending on the distribution of each variable. Categorical variables were expressed as percentages. The chi-squared test was used for comparing categorical variables, and a significance level of *p* < 0.05 was employed.

## 3. Results

A total of twenty-seven patients underwent simultaneous bilateral cochlear implantation (BiCI). The age of the patients ranged from 19 to 70 years, with a mean age of 37 ± 15.5 years. Among the participants, 59.3% were male (16/27) and 40.7% were female (11/27). Out of the total, 7.4% were revision simultaneous (BiCI) surgeries (2/27). Childhood-onset hearing loss was observed in 51.9% of the patients (14/27), while hearing loss developed later in life for 29.6% of patients (8/27); in the remaining 18.5%, the onset of hearing loss was unknown (5/27). Overall, the reported causes of hearing loss included meningitis (2, 7.4%), trauma (3, 11.1%), non-specific high-grade fever (3, 11.1%), and brucellosis infection (1, 3.7%), and was unknown for the majority of patients (18, 66.7%). A family history of hearing problems was reported by 25.9% of patients (7/27). Prior to surgery, all patients had intact tympanic membranes without active infections. Facial nerve integrity was ensured in all patients except one (3.7%). Most patients did not have any chronic illnesses (70.3%). All subjects met the criteria for CI surgery, and were diagnosed with bilateral severe to profound sensorineural hearing loss, with poor word recognition scores and minimal or no benefit from hearing aids. Computed tomography (CT) and magnetic resonance imaging (MRI) findings in (88.9%) of patients demonstrated normal morphology of the inner and middle ear, a well-pneumatized mastoid bone, and a normal cochleovestibular nerve. Labyrinthine ossificans (LO) were present in two patients (7.4%), and one patient (3.7%) had a retrofenestral otospongiosis. The mean surgical time was 254 ± 78 min, Interestingly, studying the correlation between age at implantation and surgical time revealed a significant moderate positive correlation (r = 0.48, *p*-value = 0.02). Further details of patient characteristics are presented in Table 1.

In the postoperative period, twenty-four patients (88.8%) remained clinically stable without any complaints, while three patients (11.1%) experienced mild dizziness that resolved with observation. Importantly, all patients had clean and dry surgical wounds without any discernible swelling or collections. Intraoperative complications were minimal, with only one patient experiencing bleeding from the jugular bulb, which was successfully controlled during surgery. Partial electrode array insertion occurred in two ears, while full electrode insertion was achieved in all other cases. Patients had an uneventful recovery during their hospital stay, which ranged from 0 to 2 days. Further details of the surgical findings are provided in Table 2.

During the first outpatient visit following surgery with a mean of 8.9 days, 22 patients (81.5%) expressed satisfaction with their hearing and reported no active complaints. Some patients experienced tinnitus, complained of loud noises, mild pain, changes in taste, depression of the mastoid bone, and preauricular swelling. However, no issues were encountered with the fitting of the external device, and the surgical wounds healed without swelling or signs of infection in all of our patients. Additionally, examination of the ears revealed normal tympanic membranes and intact facial nerves. The mean follow-up period was 38.6 months, The patients reported no problems, and the wounds healed well in all of them during the last follow-up. The frequency of patient complaints at the first outpatient visit is shown in Table 3.

Two notable cases in our cohort underwent revision with simultaneous BiCI, one with a history of brucellosis preceding the onset of hearing loss. This patient had previously undergone cochlear implant surgery in one ear, resulting in improved hearing. However, after a five-year period, the implanted device failed for reasons that remain unknown. Consequently, our cochlear committee decided to proceed with simultaneous BiCI. The previous electrode was completely removed, and a new electrode was successfully inserted through the round window without encountering any complications. During the recovery period, the patient experienced a brief episode of dizziness, which spontaneously resolved. Subsequently, the patient was discharged in good condition after a one-day hospital stay. The other patient, who had unilateral CI surgery at the age of 19 years, showed no benefit from CI devices; her hearing assessment showed no response in the implanted ear, with poor speech discrimination. After investigation with a CT scan, it was shown that the electrodes were condensed at the promontory, not following the cochlea turns. The decision to proceed with revision surgery with simultaneous BiCI was made. Intraoperatively, the old electrode was removed completely. However, the scala tympnai was sclerotic, and the electrode could not be inserted through the round window, so a cochleostomy was performed at the scala vestibuli, and complete electrode insertion was achieved. A minimal gusher was encountered in this patient, but it stopped with no further management. Post-surgery, the patient had a mild episode of vertigo that improved spontaneously, and was discharged the next day without any complaints.

## 4. Discussion

The present study demonstrates the feasibility of performing simultaneous BiCI in adults, challenging the traditional sequential approach. This is in contrast to pediatric patients, where early simultaneous BiCI is necessary for optimal auditory development [15]. Simultaneous BiCI carried concerns regarding a higher risk of perioperative complications and prolonged surgical time. We followed a consistent approach in simultaneous BiCI surgery that could help to decrease surgical time. For instance, facial nerve monitoring was inserted in the midline to stimulate both sides. Shaving was unnecessary in our patients and this saved a little time. In addition, we minimized the surgical incisions to around 3 cm in length, which could assist in faster wound closure and healing. We prepped and draped the patient’s ears altogether by placing a large drape behind the head to the mid-face anteriorly, so both ears were in a sterile field, thereby eliminating the need to prepare and drape each ear individually. A study showed that prolonged surgical time may be related to repeating the sterility and re-draping of the head before operating on the second ear [28]. Monopolar cautery can be used sufficiently for both ears to make skin flaps and hemostasis. However, in order to utilize monopolar cautery in simultaneous BiCI, the skin flap, pocket for the implant, mastoidectomy, and posterior tympanotomy through the facial recess were finished on one side, then the head was turned and similar steps performed on the other ear, with instruments table and microscope moved to the opposite side. Here, monopolar cautery is removed and the implantation is placed [29]. In this order, monopolar cautery can be utilized for hemostasis without posing a risk to the implant device. A study has shown that operating time decreases as the implantation team gains experience [29]. Another study has reported that the involvement of trainees in CI surgery is associated with prolonged surgical time [30]. Our findings indicate that adult patients can undergo simultaneous BiCI without significant side effects or complications. There were only three patients who experienced transient dizziness during the hospital stay, which resolved spontaneously. This aligns with the study of Das et al., which reported successful simultaneous BiCI in nine patients without major intraoperative complications. However, their study noted postoperative vertigo in four patients, with one case lasting for five months. In addition, dysgeusia and hematoma were reported in two patients [29], which was not observed in our cohort, except for one patient with a taste problem.

Furthermore, all patients in our study had a smooth recovery and were discharged shortly after surgery. We encountered one case of bleeding during posterior tympanotomy due to a high jugular bulb reaching the medial wall of the facial recess, which was effectively controlled. The surgery proceeded without further obstacles, and routine postoperative recovery was achieved. These results are consistent with a study by Gantz et al., which concluded that simultaneous BiCI in adults did not pose uncommon intraoperative complications, and did not lead to postoperative nystagmus, ataxia, or severe vertigo. The recovery period for simultaneous BiCI was comparable to that of unilateral cochlear implantation [30].

This study provides evidence supporting the feasibility of performing simultaneous BiCI in individuals who have previously undergone implantation in one ear. Our two patients who underwent revision simultaneous BiCI had neither major intra-operative complications nor significant problems in recovery period, and both were discharged home the next day after surgery.

This study emphasizes the benefits of simultaneous BiCI, particularly in relieving some of the patient burden. By opting for simultaneous implantations, patients can avoid the necessity of undergoing two separate surgeries, enduring multiple recovery periods, and attending additional follow-up visits for external device programming, as has been documented in prior studies [31]. In addition, A study by Yoshida et al. observed that the interval time between CI surgeries in the sequential method has an influence on usage time, and showed a negative correlation with speech discrimination results [14]. Notably, (70.3%) of our study subjects had a one-day hospital stay after simultaneous BiCI, eliminating the requirement for additional hospital admission and a second surgical procedure. This is particularly beneficial for patients who reside in remote areas and face financial challenges associated with long-distance travel. A study conducted by Hajr et al. in our region demonstrated that patients traveling from distant locations incur higher costs for each hospital visit related to CIs, including transportation and accommodation expenses [32]. Therefore, simultaneous BiCI may alleviate this financial burden for patients.

However, the cost-effectiveness of BiCI in adults remains uncertain. Crathorne et al. concluded that while there is a positive clinical effect of BiCI in adults, evidence regarding its cost-effectiveness is inconsistent [33]. Conversely, a recent randomized controlled trial in adults revealed the cost-effectiveness of simultaneous BiCI after 5–10 years of implant use [34]. This finding aligns with a study conducted in the United States that demonstrated the marginal financial benefit of simultaneous BiCI [35]. Although financial outcomes were not addressed in our present study, which focused primarily on the feasibility of simultaneous BiCI in adults, we believe that this simultaneous approach offers potential financial advantages.

We assert that simultaneous BiCI in adults can be safely performed in selected cases, building upon studies that have reported that bilateral ear procedures such as stapes and tympanoplasty surgeries can be performed safely when undertaken simultaneously [36,37,38]. One study concluded that simultaneous bilateral stapes surgery is safe and yields binaural hearing, while requiring a similar recovery period to unilateral surgery with a one-day hospital admission [37]. Likewise, a study by Gröger et al. reported that simultaneous BiCI in adults is safe, and had a significantly shorter total operating time, a shorter hospital stay, and comparable complications to sequential BiCI. However, each individual case must take into account the potential negative effects of the longer surgical duration of simultaneous BiCI [28]. Last but not least, as CI technology has developed and our understanding of the central auditory system has deepened, our optimization of the management of bilateral hearing loss has continued. Bilateral CI recipients in both adult and pediatric patients showed superior results to unilateral CI, including improved sound localization and improved speech perception in noisy environments [39]. This advantage continues through time, and this could have a good impact on cognitive performance in older age [28].

Overall, our study involving 27 adult patients demonstrates the feasibility of simultaneous BiCI without significant drawbacks, reducing the duration required to regain binaural hearing and minimizing the mute period. This present study has limitations, including its retrospective study design potentially affecting data collecting accuracy and the sample size included being relatively small. However, our favorable findings in this population could contribute to the literature on beneficial and safe methods for treating adults with hearing impairments. Although this study may lack a control group to compare and support the results, similar positive outcomes to ours have been reported in previous studies. Lastly, we encourage further control studies with larger sample sizes to explore the long-term outcomes and cost-effectiveness of simultaneous BiCI in adults.

## 5. Conclusions

Simultaneous BiCI is feasible in adults without major drawbacks, offering potential benefits by reducing the number of surgeries and hospital admissions compared to the sequential method. This approach allows for faster hearing rehabilitation and may alleviate the burden on patients and the healthcare system. Further research with larger sample sizes is warranted to investigate the cost-effectiveness of simultaneous BiCI in adults.

## Figures and Tables

**Table 1:** Patient demographics and characteristics.

Patient No.	Age at Implant (Years)	Sex	Onset of Hearing Loss (Ear Side)	Etiology	Comorbidity
1	19	F	Childhood (right) Age 16 years old (Left)	Fever	None
2	20	M	Unknown (bilateral)	Trauma	None
3	21	M	Childhood (bilateral)	Unknown	None
4	22	M	Unknown (bilateral)	Unknown	None
5	22	F	Childhood (bilateral)	Unknown	None
6	22	F	Childhood (bilateral)	Unknown	None
7	23	F	Childhood (bilateral)	Unknown	None
8	24	M	For 8 months (bilateral)	Unknown	Cerebral aneurysm
9	24	F	Childhood (bilateral)	Unknown	None
10	25	F	1 year (bilateral)	Meningitis	Diabetes mellitus, hypertension, celiac disease, chronic kidney disease
11	25	M	9 years (bilateral)	Trauma	None
12	29	M	Childhood (bilateral)	Unknown	None
13	29	F	Childhood (bilateral)	Unknown	None
14	34	M	Childhood (bilateral)	Fever	None
15	38	M	Childhood (bilateral)	Unknown	Usher syndrome
16	38	M	For 7 months (bilateral)	Fever	None
17	39	M	11 years (bilateral)	Trauma	Hyperthyroidism (Graves’ disease)
18	41	M	Unknown (bilateral)	Meningitis	Sickle cell anemia
19	45	M	Childhood (right) 15 years old (Left)	Unknown	None
20	47	F	Childhood (bilateral)	Unknown	None
21	48	M	Unknown (bilateral)	Unknown	None
22	49	F	Childhood (bilateral)	Unknown	None
23	56	M	Childhood (right) 10 years (Left)	Unknown	Bronchial asthma
24	57	F	25 years (bilateral)	Brucellosis	Diabetes mellitus, hypertension
25	63	M	2 years (bilateral)	Unknown	Diabetes mellitus, hypertension
26	64	M	20 years (bilateral)	Unknown	Ischemic heart disease
27	70	F	Unknown (bilateral)	Unknown	None

**Table 2:** Patients’ surgical findings.

Surgical Finding	% Cases
Displacement of sigmoid sinus	11.1%
Displacement of facial nerve	3.7%
Round window facing inferiorly	3.7%
Round window facing posteriorly	3.7%
Round window tilted and deep	3.7%
Narrow facial recess	7.4%
Labyrinthine ossificans	3.7%
Low lateral dura	3.7%
Sclerotic scala tympnai	3.7%
Cerebrospinal fluid gusher	3.7%
Facial nerve palsy	3.7%
High jugular bulb with dehiscent bone	3.7%

**Table 3:** Patient complaints at the first outpatient visit.

Complaint at 1st Visit	% Cases
Tinnitus	7.4%
Loud noises	3.7%
Mild pain	7.4%
Change of taste	3.7%
Depression on mastoid bone	3.7%
Preauricular swelling	3.7%

## Data Availability

The data are not publicly available due to the ethical approval agreement.
